# Digital Detection of Dementia (D Cubed) Trials

**DOI:** 10.1002/alz70858_099440

**Published:** 2025-12-25

**Authors:** Malaz Boustani

**Affiliations:** ^1^ Regenstrief Institute, Indianapolis, IN, USA

## Abstract

**Background:**

We developed the Passive Digital Marker (PDM) and the Quick Dementia Rating Scale (QDRS) as two scalable approaches for the early detection of dementia in primary care. The PDM leverages the widely available electronic health record (EHR) data and advances in Machine Learning algorithms to detect dementia with 80% accuracy. The QDRS is a patient‐reported outcome tool for both early detection and staging of dementia with 85% accuracy. An integrated process using these two approaches (the PDM and the QDRS) may overcome the current barriers to early dementia detection in a cost‐ and time‐efficient manner.

**Method:**

A pragmatic cluster‐randomized controlled comparative effectiveness (NIH Stage IV) trial in Nine Federally Qualified Health Centers. 5,325 patients with a mean age of 71.1 (SD=5.92), 62% female, 55% African American, and 16% of Hispanic ethnicity. Six clinics were randomized to either PDM‐only approach or to a combined approach of using both the PDM and the QDRS. Three clinics were randomized to the control group. The primary outcome measure was the incidence rate of ADRD over the subsequent 12 months as documented in the electronic health records. The study used a Generalized linear mixed models (GLMM) including fixed effects for patient and clinic level factors and a random intercept to model patients nested within clinics was used.

**Results:**

The odds of a dementia diagnosis were 41% higher in PDM+QDRS clinics than Services as Usual Clinics (OR=1.41, 95%CI: 1.18, 1.68). The odds of a dementia diagnosis were also 1% higher in PDM clinics than services as Usual but this difference was not statistically significant (OR=1.01, 95%CI: 0.82, 1.25). This intervention effect was observed even after adjusting for potential differences in patient characteristics (PDM+QDRS vs Services as Usual ‐ adjusted OR = 1.44, 95%CI: 1.19, 1.75 and PDM vs Services as Usual – adjusted OR = 1.06, 95%CI: 0.84, 1.33).

**Conclusion:**

The combined approach of using the PDM and the QDRS is an effective and scalable approach for early detection of dementia in diverse federally qualified health centers.

